# Anterior Segment Complications After Radiotherapy for Sinonasal and Nasopharyngeal Cancer: High Frequency in Maxillary Sinus Cancer Treated with RADPLAT

**DOI:** 10.3390/jcm15176862

**Published:** 2026-09-04

**Authors:** Mutsumi Koyama, Seika Den, Akinori Baba, Hirokazu Ashida, Masao Kobayashi, Tadashi Nakano

**Affiliations:** 1 Department of Ophthalmology, The Jikei University School of Medicine, 3-19-18, Nishishinbashi, Minato City 1050003, Tokyo, Japan; 2 Department of Ophthalmology, The Jikei University Katsushika Medical Center, 6-41-2, Aoto, Katsushika City 1258506, Tokyo, Japan; 3 Baba Eye Clinic, 1688 Takahayashihigashicho, Ota City 3730825, Gunma, Japan; 4 Department of Radiology, The Jikei University School of Medicine, 3-19-18, Nishishinbashi, Minato City 1050003, Tokyo, Japan

**Keywords:** head and neck cancer, intensity-modulated radiotherapy, RADPLAT, anterior segment complication, ocular toxicity, maxillary sinus cancer

## Abstract

**Objectives**: To characterize the frequency and temporal patterns of anterior segment findings after radiotherapy for sinonasal and nasopharyngeal cancer, with particular focus on anterior segment neovascularization and its association with superselective intra-arterial cisplatin infusion with concurrent radiotherapy (RADPLAT). **Methods**: We retrospectively reviewed 113 patients treated with intensity-modulated radiotherapy between 2019 and 2022. Anterior segment findings were evaluated using the whole cohort as the denominator and among patients referred for ocular symptoms. Cumulative incidence was estimated with death as a competing risk using the Aalen–Johansen estimator and compared using Gray’s test. **Results**: Median follow-up was 1666 days. Forty-seven patients (41.6%) were referred to ophthalmology. Anterior segment findings occurred in 16/60 patients (26.7%) with nasal cavity cancer and 13/31 (41.9%) with maxillary sinus cancer (*p* = 0.16); 5-year cumulative incidences were 27.7% and 46.1%, respectively (*p* = 0.018). Anterior segment neovascularization occurred in 7/113 patients (6.2%), including 6/21 (28.6%) treated with RADPLAT and 1/92 (1.1%) without RADPLAT (*p* < 0.001); 5-year cumulative incidences were 31.4% and 1.4%, respectively (*p* < 0.001). Among patients with maxillary sinus cancer, neovascularization occurred in 5/19 RADPLAT-treated patients and 0/12 non-RADPLAT patients (*p* = 0.13); however, median follow-up was substantially shorter in the non-RADPLAT group (385 vs. 1784 days). Neovascularization developed late (median, 975 days), and four affected patients had final best-corrected visual acuity below 0.05. **Conclusions**: Anterior segment findings showed distinct temporal patterns after radiotherapy. Late, vision-threatening neovascularization was strongly associated with RADPLAT, although causality cannot be established because treatment selection was confounded by tumor site, disease extent, orbital invasion, and radiotherapy characteristics. These findings identify anterior segment neovascularization as an important safety signal warranting prospective evaluation. Standardized baseline ophthalmological assessment, long-term surveillance, and patient-level ocular dosimetry are needed to clarify mechanisms and define appropriate follow-up strategies.

## 1. Introduction

Sinonasal and nasopharyngeal tumors arise in close proximity to critical ocular structures, and radiotherapy plays an important role in their treatment. When the radiation field extends toward the orbit, a range of ocular complications may occur, involving the ocular surface, eyelids, cornea, lens, retina, and optic nerve [1,2,3,4,5].

Intensity-modulated radiotherapy (IMRT) allows precise dose delivery to tumor targets while reducing exposure to adjacent normal tissues [6,7] and it has been reported to lower the rate of severe ocular toxicity compared with conventional techniques [8]. Nevertheless, ocular complications such as dry eye disease and meibomian gland dysfunction have been reported even after IMRT [2,9,10,11].

Anterior segment findings after radiotherapy may differ substantially in their timing and clinical significance [1,9,12]. Ocular surface abnormalities such as superficial punctate keratopathy and conjunctival inflammation may develop relatively early and are often manageable when detected promptly. In contrast, neovascularization of the iris or anterior chamber angle may lead to neovascular glaucoma and severe visual loss. Such neovascularization is generally associated with retinal or ocular ischemia, and its occurrence after radiotherapy may therefore represent a clinically distinct form of late ocular toxicity. However, the frequency, timing, and visual consequences of anterior segment neovascularization after radiotherapy for sinonasal tumors have not been well characterized. For maxillary sinus cancer, superselective intra-arterial cisplatin infusion with concurrent radiotherapy (RADPLAT) has been used as an organ-preserving treatment approach [13,14,15,16]. In this approach, high concentrations of cisplatin are delivered into the arterial territory supplying the tumor while radiotherapy is administered concurrently. Previous reports of RADPLAT have primarily described ocular toxicity as aggregated adverse-event categories, and detailed ophthalmological findings remain limited. In particular, whether anterior segment neovascularization represents a clinically relevant late complication in patients treated with RADPLAT has not been established.

In this retrospective cohort study, we evaluated the spectrum and temporal pattern of anterior segment findings after IMRT for sinonasal and nasopharyngeal tumors, with particular focus on anterior segment neovascularization and its association with RADPLAT. We also examined ophthalmology referral patterns and differences in anterior segment findings according to primary tumor site.

## 2. Materials and Methods

### 2.1. Study Design and Patients

This was a single-center, retrospective study. The study was conducted in accordance with the Declaration of Helsinki and approved by the Institutional Review Board of the Jikei University School of Medicine, which waived the requirement for individual informed consent given the retrospective design of the study. We included patients with sinonasal or nasopharyngeal tumors because these tumors arise in anatomical regions where radiotherapy may result in clinically relevant radiation exposure to the orbit and ocular structures. We reviewed all patients who underwent radiotherapy for these tumors at our institution between April 2019 and August 2022. Patients were classified according to the primary tumor site as follows: nasopharynx, maxillary sinus, nasal cavity (including the nasal septum and nasal vestibule), other paranasal sinuses (ethmoid, frontal, and sphenoid sinuses). Tumors of the nasal septum and nasal vestibule were grouped with nasal cavity tumors because they shared the same anatomical compartment (ICD-O topography code C30.0).

Clinical data, including age, sex, primary tumor site, TNM classification [17], radiologic orbital invasion on pretreatment computed tomography or magnetic resonance imaging, histology, and prior tumor resection and reconstruction were obtained from the patients’ medical records. Ophthalmology referral status, date of last visit at the treating department, and vital status were also obtained from the patients’ medical records. Prior tumor resection was defined as resection performed on or before the first day of radiotherapy.

Follow-up duration was defined as the interval from the start of radiotherapy to the last documented visit at the treating department, death, or the data cutoff, whichever occurred first, and was used as the time at risk for all time-to-event analyses. One patient underwent enucleation 176 days after the start of radiotherapy; because no anterior segment finding can arise thereafter, the time at risk for that patient was censored at the date of enucleation. Follow-up at the ophthalmology department was recorded separately; because ophthalmological follow-up was continued according to the severity of the ocular findings, it depends on the outcome and was therefore not used to define the time at risk.

### 2.2. Radiotherapy

All patients were treated with IMRT. Patients were classified as the RADPLAT group (IMRT with superselective intra-arterial cisplatin) or the non-RADPLAT group (IMRT without intra-arterial cisplatin), irrespective of the use of other systemic chemotherapy. Prescribed radiation dose and fractionation, cisplatin dose and number of administrations, and targeted artery were reviewed.

Prescription doses were determined by the primary site, treatment intent, and risk volume. For nasopharyngeal carcinoma, gross disease was treated to 66–70 Gy and elective low-risk volumes to 54–56 Gy. For maxillary sinus cancer, the prescribed dose differed between definitive and postoperative treatment. Tumors of the nasal cavity and the other paranasal sinuses were generally treated postoperatively, with definitive doses used when resection was not feasible. Institutional planning constraints for ocular structures were applied to the uninvolved side, with maximum doses of 54 Gy to the optic nerve and 45 Gy to the retina; the lens was constrained to 10 Gy or less where achievable. On the involved side, dose constraints to the retina and optic nerve were not applied in patients with T4 disease or in those treated with RADPLAT, because the target volume in these patients’ encompassed structures that would have been resected, including the globe, had surgery been performed. The conjunctiva was not routinely contoured as an organ at risk. The dose delivered to the ocular surface and anterior segment therefore varied between patients and was not constrained in the subgroup at highest risk of complications.

For patients treated with RADPLAT, the artery or arteries catheterized for infusion at the first session, the proportion of the cisplatin dose delivered through each, and any prophylactic embolization of branches were recorded from the interventional radiology reports.

### 2.3. Ophthalmological Assessment

Patients who developed ocular symptoms after radiotherapy were referred to the ophthalmology department for further evaluation. Ophthalmological evaluation included best corrected visual acuity, intraocular pressure, slit-lamp biomicroscopy with fluorescein vital staining and fundoscopy.

For lateralized tumors with unilateral irradiation, findings in the ipsilateral irradiated eye were recorded. For midline or bilateral tumors, or when both eyes were included in the radiation field, the eye receiving the higher radiation exposure based on the radiotherapy treatment plan was selected for analysis. Thus, one eye per patient was evaluated throughout the study. Pre-existing ocular conditions were identified from the medical records, including documented ophthalmologic history and pre-treatment consultation notes when available.

The following anterior segment findings were evaluated: superficial punctate keratopathy (SPK; punctate corneal epithelial damage identified by fluorescein staining), corneal epithelial defect (continuous epithelial loss ≥ 1 mm in diameter), conjunctivitis (conjunctival hyperemia with or without discharge), madarosis (partial or complete loss of eyelashes), blepharitis (erythema and scaling of the eyelid margin), lacrimal drainage obstruction (punctal, canalicular, or nasolacrimal duct obstruction), eyelid malposition (entropion, ectropion, or symblepharon), and anterior segment neovascularization. Anterior segment neovascularization was defined as rubeosis iridis, identified as iris neovascularization on slit-lamp examination, and/or neovascularization of the angle with neovascular glaucoma [18]; the two were recorded separately and analyzed together as the primary outcome. Cataract, radiation retinopathy, and optic neuropathy were excluded from the anterior segment analysis. Lacrimal and eyelid findings that were documented as attributable to prior surgery, tumor extension, drug effect, or another identified cause, and those recorded as pre-existing, were not counted as radiotherapy-associated findings.

Ocular findings were classified according to whether they were observed at the first post-radiotherapy (on referral date) or were detected during subsequent follow-up.

### 2.4. Outcomes

We evaluated anterior segment findings identified after radiotherapy, including their occurrence, types, and timing. Particular attention was given to anterior segment neovascularization and was additionally evaluated in relation to RADPLAT because of its clinical severity.

We also evaluated ophthalmology referral patterns and differences in anterior segment findings according to primary tumor site. Findings were classified as either present at the first post-radiotherapy ophthalmological examination or newly detected during subsequent follow-up.

The occurrence of anterior segment findings was calculated using all patients treated at each primary site as the denominator. Corresponding proportions among patients referred to ophthalmology were analyzed separately as the diagnostic yield among symptomatic, referred patients.

### 2.5. Statistical Analysis

Continuous variables are presented as mean ± standard deviation or median and range, as appropriate, and categorical variables as number and percentage. Baseline characteristics were compared across the four primary sites using the Kruskal–Wallis test for continuous variables and the chi-square test for categorical variables. Categorical variables were compared using the chi-square test or Fisher’s exact test, as appropriate.

The occurrence of anterior segment findings was compared between the nasal cavity and maxillary sinus groups using Fisher’s exact test, with all treated patients at each site as the denominator. Nasopharyngeal tumors were described separately because of their anatomical distance from the anterior segment, and patients with other paranasal sinus tumors were excluded from formal between-site comparisons because of the small sample size.

The association between RADPLAT and anterior segment neovascularization was evaluated in the whole cohort using Fisher’s exact test. Time-to-event analyses were performed from the start of radiotherapy. Cumulative incidence functions were estimated using the Aalen–Johansen estimator, with death before the outcome treated as a competing event, and groups were compared using Gray’s test. Patients who were never referred to ophthalmology were treated as event-free until death or the end of follow-up in these analyses.

Ophthalmology referral rates were compared across the four primary sites using the chi-square test. The corresponding comparison of anterior segment findings restricted to patients referred to ophthalmology was performed separately and interpreted as the diagnostic yield among symptomatic, referred patients.

Within the maxillary sinus subgroup, age and prescribed radiation dose were compared between the RADPLAT and non-RADPLAT groups using the Mann–Whitney U test, and categorical variables using Fisher’s exact test. Analyses of individual anterior segment finding types and comparisons within the maxillary sinus subgroup were considered exploratory and were not adjusted for multiple comparisons. Odds ratios (ORs) with 95% confidence intervals (CIs) were reported where applicable. All tests were two-sided, and *p* < 0.05 was considered statistically significant.

Analyses were performed using Python (version 3.12) with SciPy (version 1.17) and StatsModels (version 0.14). The Aalen–Johansen estimator and Gray’s test were implemented directly and verified to reproduce the log-rank statistic in the absence of competing events. During the preparation of this manuscript, the authors used an AI language model (Claude Opus 5, Anthropic, San Francisco, CA, USA) for the purposes of the manuscript drafting and language editing.

## 3. Results

### 3.1. Patient Characteristics

A total of 113 patients were included: 60 with nasal cavity, 31 with maxillary sinus, 19 with nasopharyngeal, and 3 with other paranasal sinus tumors. The patient characteristics by primary site are summarized in Table 1. Age differed significantly across the four primary sites (*p* = 0.03), with the maxillary sinus group being the oldest. Prior tumor resection had been performed in 59 of 113 patients (52.2%) and differed markedly by site, being most frequent in the nasal cavity group (49/60, 81.7%) and absent in the nasopharyngeal group (*p* < 0.001); reconstruction was documented in 31 of these patients. The mean prescribed radiation dose also differed significantly across the four sites (*p* < 0.001). Twenty-one patients were treated with RADPLAT: two in nasal cavity, and 19 in maxillary sinus. Median follow-up was 1666 days (4.6 years; range, 49–2642) for the whole cohort, and 1650, 1617, and 1830 days for the nasal cavity, maxillary sinus, and nasopharyngeal groups, respectively. Fifteen patients (13.3%) died during follow-up. Within the maxillary sinus group, median follow-up was markedly longer in the RADPLAT group than in the non-RADPLAT group (1784 versus 385 days).

### 3.2. Ophthalmology Referral

Forty-seven of 113 patients (41.6%) were referred to the ophthalmology department after radiotherapy because of ocular symptoms, at a median of 95 days from the start of treatment (range, 7–1059 days). The referral rate did not differ significantly among the three main primary sites (*p* = 0.09): nasal cavity, 48.3% (29/60); maxillary sinus, 45.2% (14/31); and nasopharynx, 15.8% (3/19). One of the three patients with other paranasal sinus tumors was referred.

Twelve of the 113 patients had been examined by an ophthalmologist before radiotherapy. In seven of these, the findings recorded at that examination were attributable to tumor invasion or to prior surgery, and three of the seven were also followed after radiotherapy. In these three patients the pre-treatment findings were superficial punctate keratopathy associated with postoperative lagophthalmos, tumor-related optic neuropathy, and tumor-related orbital apex syndrome, respectively.

### 3.3. Anterior Segment Findings by Primary Site

**Figure 1:**
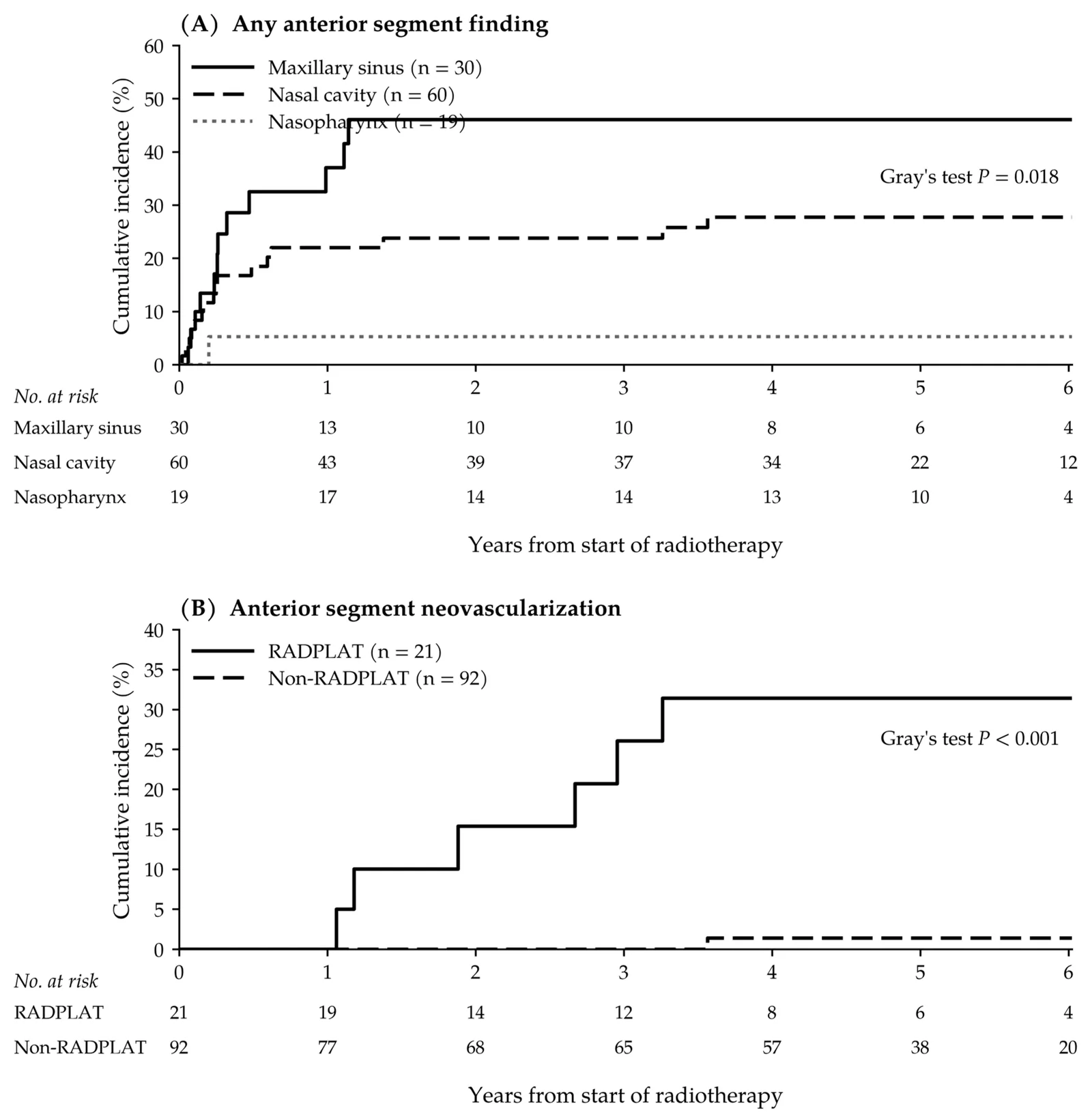
Cumulative incidence of ocular findings after radiotherapy, with death as a competing event. (**A**) Any anterior segment finding by primary site (Gray’s test, *p* = 0.018); one patient whose superficial punctate keratopathy was documented before radiotherapy was excluded from the at-risk set, and the three patients with other paranasal sinus tumors are excluded from this comparison. (**B**) Anterior segment neovascularization by the use of intra-arterial cisplatin (Gray’s test, *p* < 0.001). Numbers at risk are given below each panel.

Using the whole cohort at each primary site as the denominator, all anterior segment findings during follow-up were identified in 16 of 60 patients (26.7%) with nasal cavity cancer and in 13 of 31 patients (41.9%) with maxillary sinus cancer (Fisher’s exact test, *p* = 0.16), and in 1 of 19 patients (5.3%) with nasopharyngeal cancer (Table 2 (a)). The 5-year cumulative incidence, with death treated as a competing risk, was 27.7% for the nasal cavity group and 46.1% for the maxillary sinus group (Gray’s test, *p* = 0.018; Figure 1A). Among the 47 patients referred to ophthalmology, findings were identified in 30 (63.8%): 55.2% (16/29) for the nasal cavity, 92.9% (13/14) for the maxillary sinus, and 33.3% (1/3) for the nasopharynx (Table 2 (b)). Restricted to the nasal cavity and maxillary sinus groups, this referral-based proportion was higher in the maxillary sinus group (OR 10.56; 95% CI, 1.22–91.7; Fisher’s exact test, *p* = 0.017). Because referral was driven by ocular symptoms, this comparison is interpreted as the diagnostic yield among symptomatic, referred patients rather than as a risk comparison across the whole cohort, and the wide confidence interval is compatible with effects ranging from marginal to very large.

The most common finding overall was SPK, identified in 17 of 113 patients (15.0%) during follow-up, which was more frequent in maxillary sinus cancer than in nasal cavity cancer (*p* = 0.05). The next most common observation was conjunctivitis.

In the whole cohort, the mean prescribed radiation dose was higher in patients with anterior segment findings (67.6 ± 3.8 Gy; median, 70.0 Gy; *n* = 30) than in those without (63.9 ± 8.0 Gy; median, 66.0 Gy; *n* = 83) (Mann–Whitney U test, *p* = 0.003). The same comparison restricted to the 47 referred patients gave 67.6 ± 3.8 Gy versus 62.8 ± 6.2 Gy (*p* = 0.002). Because prescribed dose was closely related to primary site, T classification, and the use of RADPLAT, this association cannot be attributed to radiation dose alone.

### 3.4. RADPLAT vs. Non-RADPLAT

Details of the comparison between the RADPLAT and non-RADPLAT groups are shown in Table 3.

The 21 patients treated with RADPLAT differed systematically from the 92 treated without it. All had T4 disease, compared with 29 of 92 (31.5%; *p* < 0.001), and 19 (90.5%) had maxillary sinus cancer, compared with 12 of 92 (13.0%). The prescribed radiation dose was higher (70.0 ± 0.0 versus 63.7 ± 7.6 Gy; *p* < 0.001) and prior tumor resection less frequent (1 of 21 versus 58 of 92; *p* < 0.001). Follow-up was comparable between the groups (median 1719 versus 1630 days; *p* = 0.56), as was the proportion examined by an ophthalmologist (52.4% versus 39.1%; *p* = 0.33).

Anterior segment findings were identified in 11 of the 21 patients treated with RADPLAT (52.4%) and in 19 of the 92 treated without it (20.7%; *p* = 0.005). The difference was largest for corneal findings: superficial punctate keratopathy occurred in 8 (38.1%) versus 9 (9.8%; *p* = 0.003) and corneal epithelial defect in 5 (23.8%) versus 1 (1.1%; *p* < 0.001). Eyelid malposition occurred only after RADPLAT (4 of 21 versus 0 of 92; *p* < 0.001). Conjunctivitis, blepharitis, madarosis and cataract did not differ significantly between the groups.

The median cumulative cisplatin dose was 1070 mg (range, 820–1330), delivered in a median of seven intra-arterial infusions. Cisplatin was infused through a median of two arteries per patient (range 1–4). The maxillary artery was catheterized in 20 of the 21 patients (95.2%), most often its third segment (15 patients), and delivered a median of 90% of the dose (range 0–100%). The facial or transverse facial artery was used in 10 patients (47.6%) and the middle or accessory meningeal artery in 5 (23.8%); infusion was bilateral in 2. Prophylactic coil embolization of branches was performed in 5 patients and attempted without success in 1. The distribution of infused arteries did not differ between the six patients who developed anterior segment neovascularization and the fifteen who did not; the maxillary artery was used in all six and in 14 of the 15 (*p* = 1.00). Details of patient-level arterial infusion for RADPLAT are given in Supplementary Table S1.

### 3.5. Anterior Segment Neovascularization

Anterior segment neovascularization occurred in 7 of 113 patients (6.2%). Six of 21 patients (28.6%) treated with RADPLAT developed neovascularization, compared with 1 of 92 patients (1.1%) treated without RADPLAT (Fisher’s exact test, *p* < 0.001). The 5-year cumulative incidence was 31.4% in the RADPLAT group and 1.4% in the non-RADPLAT group (Gray’s test, *p* < 0.001; Table 3, Figure 1B). The median interval from the start of radiotherapy to the diagnosis was 975 days (range 387–1302). Five of 31 patients (16.1%) had maxillary sinus cancer and 2 of 60 (3.3%) had nasal cavity cancer (Fisher’s exact test, *p* = 0.043).

Rubeosis iridis was confirmed in 4 patients, all of whom had maxillary sinus cancer treated with RADPLAT (4/31, 12.9%, versus 0/60 for the nasal cavity; *p* = 0.012); the remaining three patients had neovascularization of the angle with neovascular glaucoma without visible iris rubeosis. At the time of diagnosis, intraocular pressure ranged from 17 to 41 mmHg. Best-corrected visual acuity decreased from a median of 0.4 immediately before the event to 0.1 at diagnosis. At last follow-up, three patients had no light perception, one had hand motion, and three retained measurable acuity (0.5, 0.1, and 0.07); using a threshold of best-corrected visual acuity below 0.05, four of the seven patients were blind in the affected eye, including four of the five patients with maxillary sinus cancer treated with RADPLAT. Patient-level details are given in Table 4.

Because RADPLAT was used almost exclusively for T4 maxillary sinus tumors treated to 70 Gy, this comparison cannot separate the effect of intra-arterial cisplatin from those of tumor site, stage and radiation dose. The same comparison restricted to the 31 patients with maxillary sinus cancer, in which primary site is held constant, is shown in Supplementary Table S2: anterior segment neovascularization occurred in 5 of 19 patients treated with RADPLAT (26.3%) and in none of the 12 treated without it (*p* = 0.13), with a 5-year cumulative incidence of 29.1% versus 0% (Gray’s test, *p* = 0.15). Although these differences did not reach statistical significance, every event in this subgroup occurred after RADPLAT, and observation was markedly unequal: median follow-up was 1784 days in the RADPLAT group but only 385 days in the non-RADPLAT group, shorter than the median interval to neovascularization.

### 3.6. Types and Timing of Anterior Segment Complications

**Figure 2:**
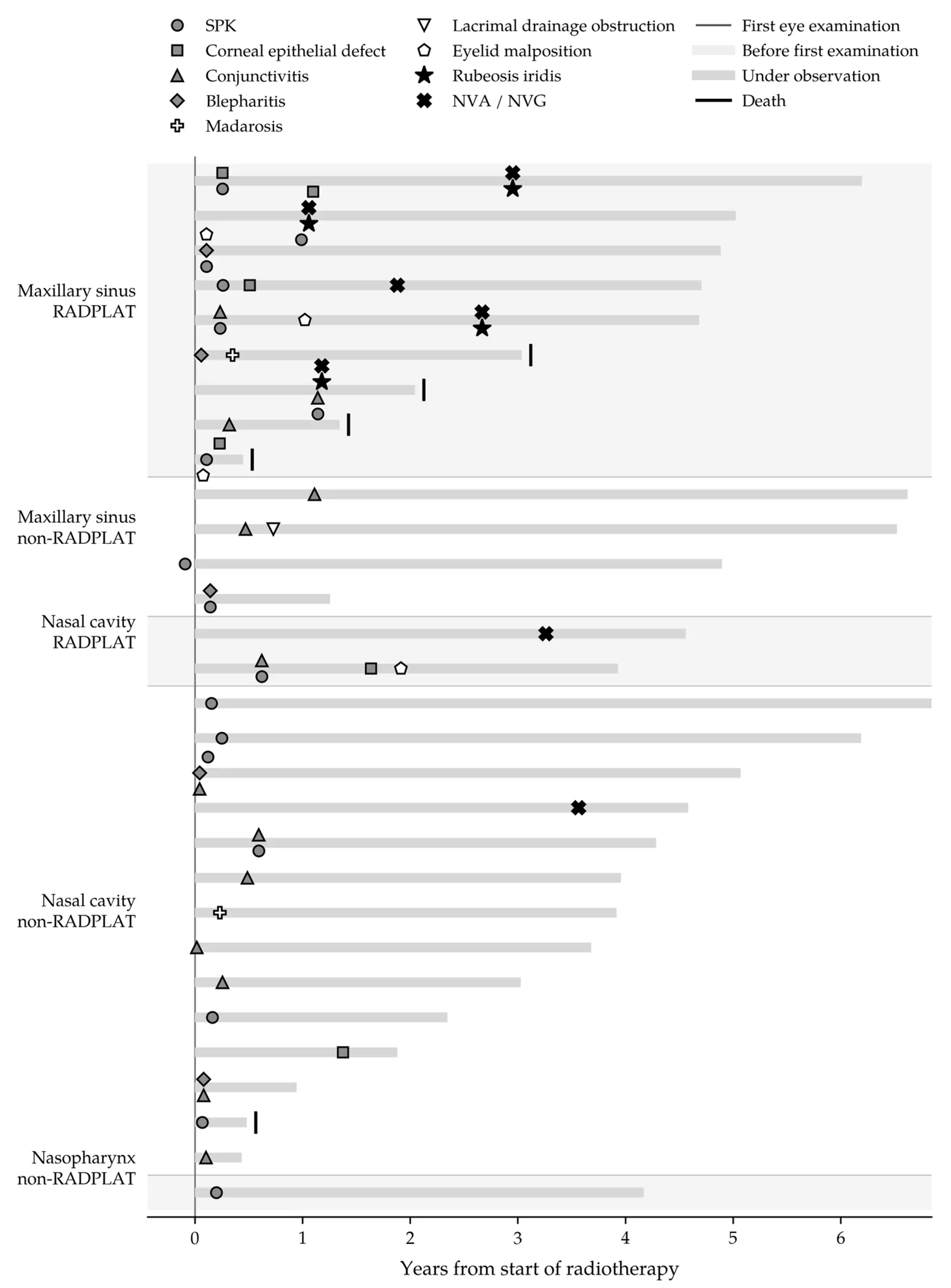
Timing of anterior segment findings in the 30 affected patients, grouped by primary site and by the use of intra-arterial cisplatin. Each bar is one patient’s follow-up from the first day of radiotherapy; the pale segment precedes the first ophthalmological examination and the darker segment is the period under ophthalmological observation, separated by a vertical line. Symbols mark the date each finding was recorded and the short vertical bar marks death. One patient had superficial punctate keratopathy documented before radiotherapy, shown to the left of time zero.

Of the 30 patients with anterior segment findings, the finding was observed at the first post-radiotherapy ophthalmological examination in 23 and was detected during subsequent follow-up in 7 (Supplementary Table S3). Ocular surface findings were predominantly observed at the first examination (superficial punctate keratopathy 14 of 17; conjunctivitis 11 of 13). All 6 patients with corneal epithelial defect had documented preceding SPK, representing progression in 6 of the 17 patients with SPK (35.3%), and median interval from the start of radiotherapy was 80 days for SPK, while 294 days for corneal epithelial defect. Anterior segment neovascularization occurred substantially later, with a median interval of 975 days (range, 387–1302). The follow-up duration of individual patients and the timing of each finding are shown in Figure 2.

## 4. Discussion

In this study, we reviewed all consecutive patients who received radiotherapy for sinonasal or nasopharyngeal cancer, and we then analyzed those who were referred to ophthalmology because of ocular symptoms. The referral rate did not differ significantly among the four primary sites and was numerically highest in patients with nasal cavity cancer. Because anterior segment complications tend to appear early and are of direct clinical relevance, we focused on these complications in the present analysis. Nasopharyngeal cancer is anatomically distant from the anterior segment and rarely causes such complications, and we therefore compared the anterior segment complication rate between nasal cavity cancer and maxillary sinus cancer.

SPK was the most common complication and was more frequent in maxillary sinus cancer than in nasal cavity cancer. SPK tended to appear early (median, 80 days), whereas corneal epithelial defect appeared later (median, 294 days) and all six corneal epithelial defects developed in eyes with preceding SPK. SPK is a mild finding in itself, but it reflects ocular surface breakdown and may progress to corneal epithelial defect and ulceration if left unmanaged [9,19]. Detection while the findings are still mild is therefore clinically important.

In contrast, anterior segment neovascularization was clinically distinct from the ocular surface findings because of its delayed onset and severe visual consequences. All seven neovascular events were detected during subsequent follow-up rather than at the first post-radiotherapy ophthalmological examination, with a median interval of 975 days (range, 387–1302 days) from the start of radiotherapy. This temporal separation was also apparent in the swimmer plot, in which ocular surface findings clustered relatively early whereas neovascular events emerged approximately 1–3.5 years after treatment.

Anterior segment neovascularization was strongly associated with RADPLAT. It occurred in 6 of 21 patients treated with RADPLAT and in 1 of 92 patients treated without it, and the difference persisted when death was treated as a competing risk (5-year cumulative incidence, 31.4% versus 1.4%; Gray’s test, *p* < 0.001). The neovascular events were associated with substantial visual morbidity: four of the seven affected patients had a final best-corrected visual acuity below 0.05, including three with no light perception.

All four patients with confirmed rubeosis iridis had maxillary sinus cancer treated with RADPLAT. A frequency comparable to that observed here has not previously been described after radiotherapy for maxillary sinus cancer. In the affected patients, diabetes mellitus, diabetic retinopathy, carotid imaging abnormalities or clinical signs of ocular ischemic syndrome, and previous retinal vein occlusion were all absent, and no retinal abnormality had been documented at the examinations preceding the neovascular event; at diagnosis the fundus could not be examined because of hyphema.

In RADPLAT, cisplatin is infused at high concentration into the feeding artery of the tumor, and vascular complications within the perfused territory have been reported since the original description of the technique [20,21,22]. In maxillary sinus cancer, the maxillary artery is a major arterial route used for intra-arterial cisplatin delivery and has anastomotic connections with the ophthalmic arterial system; ischemic injury to the anterior segment vasculature is one possible mechanism. However, the maxillary artery was infused in nearly all RADPLAT-treated patients regardless of whether neovascularization occurred, so the arterial territory alone does not identify a distinct route of exposure; no vascular imaging or treatment-territory analysis was performed to test this hypothesis directly, and it should be regarded as speculative.

The strong association between RADPLAT and anterior segment neovascularization should nevertheless be interpreted cautiously because RADPLAT use was closely linked to tumor site, disease extent, and radiotherapy characteristics. All 21 patients treated with RADPLAT had T4 disease, 19 had maxillary sinus cancer, and all received 70 Gy, whereas prior tumor resection was substantially less frequent in the RADPLAT group. Among patients with maxillary sinus cancer, tumor orbital invasion was also more frequent in the RADPLAT group than in the non-RADPLAT group (63.2% vs. 25.0%), although the difference was not statistically significant. Consequently, the observed association cannot distinguish the effect of intra-arterial cisplatin from those of advanced local disease, orbital proximity, higher ocular radiation exposure, or other treatment-related factors. Restriction of the comparison to maxillary sinus cancer partially addressed confounding by primary site: neovascularization occurred in 5 of 19 RADPLAT-treated patients and in none of 12 non-RADPLAT patients, although the difference was not statistically significant. Interpretation of this subgroup comparison is limited by markedly unequal observation time, because median follow-up was 1784 days in the RADPLAT group but only 385 days in the non-RADPLAT group, substantially shorter than the median 975-day interval to neovascularization. Thus, the whole-cohort and maxillary-sinus-only comparisons have complementary limitations: the former is strongly confounded by treatment selection, whereas the latter is constrained by small numbers and unequal follow-up. The present findings should therefore be regarded as a clinically important association and safety signal rather than evidence that intra-arterial cisplatin itself causes anterior segment neovascularization.

The proportion of patients with anterior segment findings among those referred to ophthalmology was higher in maxillary sinus cancer than in nasal cavity cancer, whereas the referral rate itself was numerically highest in nasal cavity cancer. When the whole cohort was used as the denominator, this difference was no longer statistically significant, although the cumulative incidence functions differed across primary sites after accounting for follow-up and death as a competing risk. The higher referral-based proportion in maxillary sinus cancer therefore cannot be interpreted as a direct estimate of underlying risk because it is conditioned on symptom-driven ophthalmological referral. Anatomical proximity between the primary tumor and the orbit remains a plausible contributor to the observed pattern: the orbital floor forms the roof of the maxillary sinus, so that the eyelids and the ocular surface lie immediately above the target volume, and orbital invasion was present in 15 of 31 patients (48.4%) with maxillary sinus cancer. Nasal cavity tumors are separated from the orbit only by the lamina papyracea, but the exposed structures are more medial and the ocular surface is less consistently included in the high-dose region. Across the primary sites, radiation dose and the frequency of prior tumor resection varied inversely. The nasopharyngeal group received the highest mean dose (68.4 Gy) but had the lowest frequency of anterior segment findings (5.3%), which argues against prescribed radiation dose as the dominant determinant and is more consistent with anatomical proximity to the orbit, and with the ocular dose constraints applied, as the principal drivers. Primary site therefore bundles together orbital proximity, resectability, and radiation dose; in this cohort, RADPLAT use was almost entirely confined to the maxillary sinus group (19 of 21 RADPLAT-treated patients), illustrating the extent to which these factors were confounded.

In the present study, ophthalmologic referral was based on ocular symptoms, at a median of 95 days and as early as 7 days after the start of treatment; findings referred within the first one to two weeks may reflect acute chemoradiation-related mucosal toxicity rather than a distinct radiation-induced ocular complication. Ocular surface findings were largely already present at the first examination, whereas every neovascular event developed later during follow-up. Referral triggered by symptoms may therefore both delay the recognition of early ocular surface disease and miss late neovascular events until vision has already been lost. Systematic ophthalmologic follow-up was not evaluated in this study, since no patient was followed on a fixed schedule; we therefore propose it as a hypothesis for future evaluation rather than as a validated recommendation. Based on the observed timing of the findings, a provisional schedule could include ophthalmologic assessment at approximately 1 and 3 months after the start of radiotherapy to detect early ocular surface disease, and at least annual assessment thereafter, continuing beyond 3 years, for patients treated with RADPLAT for maxillary sinus cancer, given the late onset of neovascular events in this group. Such a schedule requires prospective validation.

### Limitations

This study has several limitations that constrain both estimation of the frequency of ocular findings and causal interpretation of the association with RADPLAT. First, ophthalmological assessment was not systematic but was performed according to clinical referral. Asymptomatic or subclinical findings could therefore have been missed, and patients who were never referred were treated as event-free in the cumulative incidence analyses. Consequently, the reported frequencies and cumulative incidence estimates, particularly for neovascularization, should be regarded as lower-bound estimates of the true incidence. In addition, only 12 of 113 patients had a documented ophthalmological examination before radiotherapy. For common findings such as superficial punctate keratopathy, blepharitis, and conjunctival disease, absence of a documented pre-treatment finding cannot be assumed to represent a normal baseline, creating a potential source of misattribution as well as under-ascertainment. Second, patient-level dose-volume histogram data for ocular structures were unavailable. Total prescribed dose is an inadequate surrogate for the actual dose received by the globe, cornea, conjunctiva, lacrimal gland, retina, or optic nerve, and the contribution of radiation exposure to individual outcomes could not be quantified. Third, RADPLAT use was strongly confounded by primary site, T classification, orbital invasion, radiation dose, and prior surgery. With only seven neovascular events, these interrelated factors could not be reliably separated in an adjusted model, and no causal effect of RADPLAT can be estimated from these data. Fourth, the number of neovascular events and the size of the maxillary sinus non-RADPLAT comparator group were small, and follow-up within the maxillary sinus subgroup was markedly shorter in the non-RADPLAT group. The absence of neovascular events in that subgroup therefore should not be interpreted as absence of risk. Finally, analyses of individual anterior segment findings were exploratory and involved multiple comparisons without adjustment for multiplicity. These findings should therefore be considered hypothesis-generating and require confirmation in larger cohorts with standardized baseline ophthalmological assessment, systematic long-term follow-up, and patient-level ocular dosimetry.

## 5. Conclusions

Anterior segment findings after radiotherapy for sinonasal and nasopharyngeal cancer showed distinct temporal and clinical patterns, with ocular surface abnormalities occurring relatively early and neovascularization emerging as a late, vision-threatening event. Anterior segment neovascularization was strongly associated with RADPLAT and remained so after accounting for death as a competing risk. However, because RADPLAT use was closely confounded by tumor site, disease extent, orbital invasion, and radiotherapy characteristics, causality cannot be established. These findings identify late anterior segment neovascularization as a clinically important safety signal after RADPLAT and support prospective studies with standardized ophthalmological surveillance and patient-level ocular dosimetry.

## Figures and Tables

**Table 1:** Patient, tumor and treatment characteristics by primary site (*n* = 113).

Characteristic	Nasal Cavity	Maxillary Sinus	Nasopharynx	Other Paranasal Sinus	Total	*p* *
*n*	60	31	19	3	113	
Age, years, median (range)	57 (22–80)	64 (31–87)	54 (12–79)	49 (33–63)	59 (12–87)	0.03
Male sex, *n* (%)	36 (60.0)	23 (74.2)	13 (68.4)	1 (33.3)	73 (64.6)	0.36
Laterality of the primary tumor						
Unilateral, *n* (%)	55 (91.7)	31 (100.0)	7 (36.8)	2 (66.7)	95 (84.1)	
Bilateral or midline, *n* (%)	5 (8.3)	0 (0.0)	12 (63.2)	1 (33.3)	18 (15.9)	
Tumor (T) classification						
T1–T3, *n* (%)	19 (31.7)	6 (19.4)	15 (78.9)	2 (66.7)	42 (37.2)	
T4, *n* (%)	20 (33.3)	25 (80.6)	4 (21.1)	1 (33.3)	50 (44.2)	
Not assessable/not recorded, *n* (%)	21 (35.0)	0 (0.0)	0 (0.0)	0 (0.0)	21 (18.6)	
Nodal (*N*) classification						
N0, *n* (%)	23 (38.3)	24 (77.4)	1 (5.3)	2 (66.7)	50 (44.2)	
N1–N3, *n* (%)	7 (11.7)	6 (19.4)	18 (94.7)	0 (0.0)	31 (27.4)	
Nx/not recorded, *n* (%)	30 (50.0)	1 (3.2)	0 (0.0)	1 (33.3)	32 (28.3)	
Histology						
Squamous cell carcinoma, *n* (%)	27 (45.0)	29 (93.5)	15 (78.9)	3 (100.0)	74 (65.5)	
Olfactory neuroblastoma, *n* (%)	20 (33.3)	0 (0.0)	0 (0.0)	0 (0.0)	20 (17.7)	
Lymphoepithelial carcinoma, *n* (%)	0 (0.0)	0 (0.0)	4 (21.1)	0 (0.0)	4 (3.5)	
Adenoid cystic carcinoma, *n* (%)	4 (6.7)	0 (0.0)	0 (0.0)	0 (0.0)	4 (3.5)	
Adenocarcinoma, *n* (%)	3 (5.0)	0 (0.0)	0 (0.0)	0 (0.0)	3 (2.7)	
Neuroendocrine carcinoma, *n* (%)	3 (5.0)	0 (0.0)	0 (0.0)	0 (0.0)	3 (2.7)	
Mucosal melanoma, *n* (%)	1 (1.7)	0 (0.0)	0 (0.0)	0 (0.0)	1 (0.9)	
Other, *n* (%)	2 (3.3)	1 (3.2)	0 (0.0)	0 (0.0)	3 (2.7)	
Unclassifiable, *n* (%)	0 (0.0)	1 (3.2)	0 (0.0)	0 (0.0)	1 (0.9)	
Prior tumor resection, *n* (%)	49 (81.7)	9 (29.0)	0 (0.0)	1 (33.3)	59 (52.2)	<0.001
Treatment intent						
Definitive, *n* (%)	9 (15.0)	22 (71.0)	19 (100.0)	2 (66.7)	52 (46.0)	
Postoperative adjuvant, *n* (%)	49 (81.7)	8 (25.8)	0 (0.0)	1 (33.3)	58 (51.3)	
Palliative or discontinued, *n* (%)	2 (3.3)	1 (3.2)	0 (0.0)	0 (0.0)	3 (2.7)	
Radiation dose, Gy, mean ± SD	63.0 ± 7.4	66.1 ± 8.4	68.4 ± 2.2	66.7 ± 5.8	64.9 ± 7.3	<0.001
Radiation dose, Gy, median (range)	66.0 (26–70.0)	70.0 (32–70.0)	68.8 (59–71.0)	70.0 (60–70.0)	66.0 (26–71.0)	
Intra-arterial cisplatin (RADPLAT), *n* (%)	2 (3.3)	19 (61.3)	0 (0.0)	0 (0.0)	21 (18.6)	
Follow-up, days, median (range)	1650 (72–2642)	1617 (49–2439)	1830 (342–2488)	444 (163–1860)	1666 (49–2642)	
Death during follow-up, *n* (%)	3 (5.0)	6 (19.4)	4 (21.1)	2 (66.7)	15 (13.3)	
Examination on ophthalmology after radiotherapy, *n* (%)	29 (48.3)	14 (45.2)	3 (15.8)	1 (33.3)	47 (41.6)	0.09

* Kruskal–Wallis test for continuous variables and chi-square test for categorical variables, across the four primary sites. Percentages are column percentages within each primary site. Nodal stage was not recorded for 32 patients, most of whom underwent primary resection with postoperative radiotherapy. Tumor (T) classification was not assessable or not recorded in 21 patients, mainly olfactory neuroblastomas staged by the Kadish system. Three patients did not complete the planned course (32 Gy, discontinued after cerebral infarction; 26 Gy, discontinued because of Pseudomonas skull-base osteomyelitis and pachymeningitis; 45 Gy, palliative intent with priority given to treatment of a synchronous rectal cancer). These three account for the lower end of the dose range.

**Table 2:** (**a**) All anterior segment findings identified after radiotherapy, by primary site—whole-cohort denominators (*n* = 113). (**b**) The same findings among the 47 patients examined by an ophthalmologist after radiotherapy.

(**a**)							
**Finding**	**Nasal Cavity**	**Maxillary Sinus**	**Nasopharynx**	**Other Paranasal Sinus**	**Total**	***p* †**	**Onset, Days, Median (Range)**
Any anterior segment finding	16/60 (26.7)	13/31 (41.9)	1/19 (5.3)	0/3 (0.0)	30/113 (26.5)	0.16	91 (7–1302)
Superficial punctate keratopathy	7/60 (11.7)	9/31 (29.0)	1/19 (5.3)	0/3 (0.0)	17/113 (15.0)	0.05	80 (25–417)
Corneal epithelial defect	2/60 (3.3)	4/31 (12.9)	0/19 (0.0)	0/3 (0.0)	6/113 (5.3)	0.18	294 (84–598)
Conjunctivitis	8/60 (13.3)	5/31 (16.1)	0/19 (0.0)	0/3 (0.0)	13/113 (11.5)	0.76	117 (7–417)
Blepharitis	2/60 (3.3)	3/31 (9.7)	0/19 (0.0)	0/3 (0.0)	5/113 (4.4)	0.33	30 (16–52)
Madarosis	1/60 (1.7)	1/31 (3.2)	0/19 (0.0)	0/3 (0.0)	2/113 (1.8)	1.00	106 (85–128)
Eyelid malposition	1/60 (1.7)	3/31 (9.7)	0/19 (0.0)	0/3 (0.0)	4/113 (3.5)	0.11	206 (28–699)
Lacrimal drainage obstruction	0/60 (0.0)	1/31 (3.2)	0/19 (0.0)	0/3 (0.0)	1/113 (0.9)	0.34	266
Anterior segment neovascularization	2/60 (3.3)	5/31 (16.1)	0/19 (0.0)	0/3 (0.0)	7/113 (6.2)	0.04	975 (387–1302)
Rubeosis iridis	0/60 (0.0)	4/31 (12.9)	0/19 (0.0)	0/3 (0.0)	4/113 (3.5)	0.01	—
Neovascularization of the angle/neovascular glaucoma	2/60 (3.3)	5/31 (16.1)	0/19 (0.0)	0/3 (0.0)	7/113 (6.2)	0.04	—
(**b**)							
**Finding**	**Nasal Cavity**	**Maxillary Sinus**	**Nasopharynx**	**Other Paranasal Sinus**	**All Examined**		
Any anterior segment finding	16/29 (55.2)	13/14 (92.9)	1/3 (33.3)	0/1 (0.0)	30/47 (63.8)		
Superficial punctate keratopathy	7/29 (24.1)	9/14 (64.3)	1/3 (33.3)	0/1 (0.0)	17/47 (36.2)		
Corneal epithelial defect	2/29 (6.9)	4/14 (28.6)	0/3 (0.0)	0/1 (0.0)	6/47 (12.8)		
Conjunctivitis	8/29 (27.6)	5/14 (35.7)	0/3 (0.0)	0/1 (0.0)	13/47 (27.7)		
Blepharitis	2/29 (6.9)	3/14 (21.4)	0/3 (0.0)	0/1 (0.0)	5/47 (10.6)		
Madarosis	1/29 (3.4)	1/14 (7.1)	0/3 (0.0)	0/1 (0.0)	2/47 (4.3)		
Eyelid malposition	1/29 (3.4)	3/14 (21.4)	0/3 (0.0)	0/1 (0.0)	4/47 (8.5)		
Lacrimal drainage obstruction	0/29 (0.0)	1/14 (7.1)	0/3 (0.0)	0/1 (0.0)	1/47 (2.1)		
Anterior segment neovascularization	2/29 (6.9)	5/14 (35.7)	0/3 (0.0)	0/1 (0.0)	7/47 (14.9)		
Rubeosis iridis	0/29 (0.0)	4/14 (28.6)	0/3 (0.0)	0/1 (0.0)	4/47 (8.5)		
Neovascularization of the angle/neovascular glaucoma	2/29 (6.9)	5/14 (35.7)	0/3 (0.0)	0/1 (0.0)	7/47 (14.9)		

† Fisher’s exact test, maxillary sinus versus nasal cavity. Values are *n*/*N* (%) with the whole cohort treated at each site as the denominator. Onset is measured from the first day of radiotherapy. In 23 of the 30 patients, the findings were observed at the first post-radiotherapy examination. Eyelid malposition comprised lower-lid entropion in two patients, lower-lid retraction in one and symblepharon of the lower lid in one; lacrimal drainage obstruction was punctal occlusion in one patient. Rubeosis iridis and neovascularization of the angle/neovascular glaucoma are subcategories of anterior segment neovascularization and are not mutually exclusive. One patient had superficial punctate keratopathy documented before radiotherapy in association with postoperative lagophthalmos. This patient is included in the proportions above but was excluded from the at-risk set for the cumulative incidence analysis (Figure 1A) and from the onset statistics, because a finding present at the start of radiotherapy is prevalent rather than incident. Values are *n*/*N* (%) with examined patients as the denominator. These proportions describe the yield of examination among patients who reached an ophthalmologist and are not risk estimates for the treated population.

**Table 3:** Treatment, tumor characteristics and ocular findings by the use of intra-arterial cisplatin: RADPLAT versus non-RADPLAT (*n* = 113).

Variable	RADPLAT	Non-RADPLAT	*p*
*n*	21	92	
Age, years, median (range)	61 (31–79)	58 (12–87)	0.36
Male sex, *n* (%)	12 (57.1)	61 (66.3)	0.46
Primary site, *n* (%)			
Maxillary sinus	19 (90.5)	12 (13.0)	
Nasal cavity	2 (9.5)	58 (63.0)	
Nasopharynx	0 (0.0)	19 (20.7)	
Other paranasal sinuses	0 (0.0)	3 (3.3)	
T4 disease, *n* (%)	21 (100.0)	29 (31.5)	<0.001
Prior tumor resection, *n* (%)	1 (4.8)	58 (63.0)	<0.001
Radiation dose, Gy, mean ± SD	70.0 ± 0.0	63.7 ± 7.6	<0.001
Cisplatin infusions, *n*, median (range)	7 (6–7)	Not applicable	—
Cumulative cisplatin dose, mg, median (range)	1070 (820–1330)	Not applicable	—
Number of arteries infused, median (range)	2 (1–4)	Not applicable	—
Maxillary artery infused, *n* (%)	20 (95.2)	Not applicable	—
Middle or accessory meningeal artery infused, *n* (%)	5 (23.8)	Not applicable	—
Prophylactic embolization, *n* (%)	5 (23.8)	Not applicable	—
Follow-up, days, median (range)	1719 (164–2439)	1630 (49–2642)	0.56
Death during follow-up, *n* (%)	5 (23.8)	10 (10.9)	0.15
Examined by an ophthalmologist after radiotherapy, *n* (%)	11 (52.4)	36 (39.1)	0.33
Any anterior segment finding, *n* (%)	11 (52.4)	19 (20.7)	0.005
Superficial punctate keratopathy	8 (38.1)	9 (9.8)	0.003
Corneal epithelial defect	5 (23.8)	1 (1.1)	<0.001
Conjunctivitis	4 (19.0)	9 (9.8)	0.26
Blepharitis	2 (9.5)	3 (3.3)	0.23
Madarosis	1 (4.8)	1 (1.1)	0.34
Eyelid malposition	4 (19.0)	0 (0.0)	<0.001
Lacrimal drainage obstruction	0 (0.0)	1 (1.1)	1.00
Anterior segment neovascularization, *n* (%)	6 (28.6)	1 (1.1)	<0.001
Rubeosis iridis	4 (19.0)	0 (0.0)	<0.001
Neovascularization of the angle/neovascular glaucoma	6 (28.6)	1 (1.1)	<0.001
5-year cumulative incidence of neovascularization, %	31.4	1.4	<0.001 §

§ Gray test with death as a competing risk. *p* values are from the Fisher exact test unless otherwise stated; continuous variables were compared with the Mann–Whitney U test. Follow-up was measured from the first day of radiotherapy to death or the last visit to the treating department. RADPLAT denotes treatment with intra-arterial cisplatin and non-RADPLAT its absence. Concurrent systemic chemotherapy was outside the scope of this study and was not systematically recorded; residual confounding by systemic treatment cannot be excluded. Primary site, T classification, radiation dose and prior resection differed markedly between the groups, so the comparison of ocular findings is confounded by these factors; the corresponding comparison restricted to maxillary sinus cancer, in which site is held constant, is given in Supplementary Table S2. Radiologic orbital invasion, assessed only in patients with maxillary sinus or nasal cavity tumors, is reported in Supplementary Table S2. The third segment of the maxillary artery was infused in 15 patients, the second segment in 3, and the segment was not specified in 1; the maxillary artery delivered a median of 90% of the cisplatin dose (range 0–100%). The facial or transverse facial artery was infused in 10 patients and infusion was bilateral in 2. Arterial categories are not mutually exclusive. Prophylactic embolization was attempted without success in one further patient. Patient-level arterial data are given in Supplementary Table S1.

**Table 4:** Case-level details for the seven patients with anterior segment neovascularization.

Case	Primary Site	T	Orbital Invasion	RADPLAT	Dose (Gy)	Infusions	Cisplatin (mg)	Rubeosis Iridis	NVA/NVG	Onset (Days)	IOP (mmHg)	BCVA Before	BCVA at Onset	BCVA at Last Visit	Follow-Up (Days)
1	Maxillary sinus	T4b	Yes	Yes	70	7	994	Yes	Yes	387	34	0.4	0.1	NLP	1835
2	Maxillary sinus	T4a	Yes	Yes	70	7	1154	Yes	Yes	431	41	0.3	0.1	0.1	747
3	Maxillary sinus	T4a	Yes	Yes	70	7	1172	No	Yes	687	32	1.2	0.1	HM	1719
4	Maxillary sinus	T4a	Yes	Yes	70	7	988	Yes	Yes	975	24	HM	HM	NLP	1711
5	Maxillary sinus	T4a	Yes	Yes	70	7	1214	Yes	Yes	1079	27	0.6	0.1	NLP	2264
6	Nasal cavity	T4a	Yes	Yes	70	7	1088	No	Yes	1191	28	0.2	0.2	0.07	1666
7	Nasal cavity	T4b	Yes	No	66	—	—	No	Yes	1302	17	0.7	0.7	0.5	1674

BCVA, best-corrected visual acuity (decimal); IOP, intraocular pressure at the diagnosis of neovascularization; NVA, neovascularization of the angle; NVG, neovascular glaucoma; NLP, no light perception; HM, hand motion. Onset is the interval from the first day of radiotherapy to the diagnosis of neovascularization. Percentages after each artery are the share of the cisplatin dose delivered through that vessel at the first infusion. Blindness was defined as a best-corrected visual acuity below 0.05; four of the seven patients met this threshold at the last visit. Diabetes mellitus, diabetic retinopathy, carotid disease or signs of ocular ischemic syndrome, and prior retinal vein occlusion were absent in all seven. No retinal abnormality was documented at the preceding examinations, and at diagnosis the fundus could not be examined because of hyphema. The single exception was case 7, the only affected patient not treated with RADPLAT, in whom cotton-wool spots suggestive of radiation retinopathy preceded the event by eight months.

## Data Availability

The data that support the findings of this study are not publicly available due to patient privacy restrictions but are available from the corresponding author (SD) upon reasonable request.

## Supplementary Materials

The following supporting information can be downloaded at: https://www.mdpi.com/article/10.3390/jcm15176862/s1, Table S1: Patient-level arterial infusion data for the 21 patients treated with RADPLAT; Table S2. Maxillary sinus cancer (*n* = 31): RADPLAT versus non-RADPLAT, with primary site held constant; Table S3. Anterior segment findings observed at the first post-radiotherapy ophthalmological examination versus those detected during subsequent follow-up.
