# Supplementary material for: Anterior Segment Complications After Radiotherapy for Sinonasal and Nasopharyngeal Cancer: High Frequency in Maxillary Sinus Cancer Treated with RADPLAT
Source: J Clin Med. 2026 Sep 4;15(17):6862. doi: 10.3390/jcm15176862 (PMC13566033; doi:10.3390/jcm15176862)
Supplement: Supplementary file 1 [file jcm-15-06862-s001.zip › jcm-4478386-supplementary.pdf]

Supplementary Table S1. Patient-level arterial infusion data for the 21 patients treated with RADPLAT.

Case	Primary site	Side infused	Arteries infused and share of the cisplatin dose	Prophylactic embolization	Notes	Neovascularization
1	Maxillary sinus	Right	Maxillary artery third segment 40%; transverse facial artery 50%; facial artery 10%	No	—	No
2	Maxillary sinus	Left	Maxillary artery third segment 90%; transverse facial artery 10%	No	—	Yes
3	Maxillary sinus	Right	Maxillary artery third segment 100%	No	—	No
4	Maxillary sinus	Right	Maxillary artery third segment 80%; accessory meningeal artery 20%	No	—	No
5	Maxillary sinus	Left	Maxillary artery third segment 70%; transverse facial artery 20%; facial artery 10%	No	—	No
6	Maxillary sinus	Left	Maxillary artery third segment 100%	No	100% inferred because only this artery received cisplatin.	No
7	Maxillary sinus	Left	Maxillary artery second segment 50%; maxillary artery third segment 40%; middle meningeal artery 10%	Yes	Coil embolization of middle deep temporal artery and distal middle meningeal artery.	No
8	Maxillary sinus	Left	Maxillary artery third segment 80%; distal facial artery 10%; transverse facial artery 10%	No	—	Yes
9	Maxillary sinus	Left	Maxillary artery third segment 90%; facial artery 10%	No	—	Yes
10	Maxillary sinus	Right	Maxillary artery third segment 100%	No	—	No
11	Maxillary sinus	Bilateral	Left descending palatine artery 5%; right maxillary artery third segment 80%; right accessory meningeal artery 5%; right ascending palatine artery 10%	Yes	Multiple embolizations: 10% for ascending palatine artery inferred from remaining dose.	No
12	Maxillary sinus	Right	Maxillary artery third segment 100%	No	—	No
13	Maxillary sinus	Left	Middle meningeal artery 10%; maxillary artery third segment 90%	Attempted	Coil was retrieved; distal middle meningeal artery subsequently not visualized due to spasm/occlusion.	Yes
14	Maxillary sinus	Right	Maxillary artery second segment 90%; accessory meningeal artery 10%	No	—	No
15	Maxillary sinus	Left	Maxillary artery third segment 100%	No	—	No
16	Maxillary sinus	Right	External carotid artery proximal to the superficial temporal branch 80%; superior labial artery 10%; distal facial artery 5%; ascending pharyngeal artery 5%	Yes	Middle deep temporal artery and superficial temporal artery coil embolization.	No
17	Maxillary sinus	Left	Maxillary artery second segment 100%	Yes	Middle deep temporal artery coil embolization.	No
18	Maxillary sinus	Right	Maxillary artery 70%; facial artery 30%	No	—	Yes
19	Maxillary sinus	Left	Maxillary artery third segment 90%; facial artery 10%	No	—	No
20	Nasal cavity	Right	Maxillary artery second segment 80%; facial artery 10%; ascending palatine artery 5%; ascending pharyngeal artery 5%	Yes	Middle deep temporal artery coil embolization with two 2 mm × 8 cm coils.	No
21	Nasal cavity	Bilateral	Left maxillary artery 70%; left facial artery 20%; right facial artery 10%	No	—	Yes

Percentages are the share of the cisplatin dose delivered through each vessel at the first infusion session. Cases 1 to 19 had maxillary sinus cancer and cases 20 and 21 nasal cavity cancer; the case numbers are independent of those in Table 4. Prophylactic embolization was recorded as attempted when a coil was deployed but the intended vessel was not occluded.

The maxillary artery was infused in 20 of the 21 patients, the facial or transverse facial artery in 10, and the middle or accessory meningeal artery in 5; infusion was bilateral in 2. The distribution of infused arteries did not differ between the six patients who developed anterior segment neovascularization and the fifteen who did not.

Supplementary Table S2. Maxillary sinus cancer (n = 31): RADPLAT versus non-RADPLAT, with primary site held constant.

Variable	RADPLAT	Non-RADPLAT	P
n	19	12	
Age, years, median (range)	61 (31–79)	70 (52–87)	0.11
Male sex, n (%)	12 (63.2)	11 (91.7)	0.11
T4 disease, n (%)	19 (100.0)	6 (50.0)	0.001
Radiologic orbital invasion, n (%)	12 (63.2)	3 (25.0)	0.07
Prior tumor resection, n (%)	1 (5.3)	8 (66.7)	<0.001
Radiation dose, Gy, mean ± SD	70.0 ± 0.0	59.9 ± 11.2	<0.001
Cisplatin infusions, n, median (range)	7 (6–7)	Not applicable	—
Cumulative cisplatin dose, mg, median (range)	1070 (820–1330)	Not applicable	—
Number of arteries infused, median (range)	2 (1–4)	Not applicable	—
Maxillary artery infused, n (%)	18 (94.7)	Not applicable	—
Middle or accessory meningeal artery infused, n (%)	5 (26.3)	Not applicable	—
Prophylactic embolization, n (%)	4 (21.1)	Not applicable	—
Follow-up, days, median (range)	1784 (164–2439)	385 (49–2418)	0.03
Death during follow-up, n (%)	5 (26.3)	1 (8.3)	0.36
Examined by an ophthalmologist after radiotherapy, n (%)	9 (47.4)	5 (41.7)	1.00
Any anterior segment finding, n (%)	9 (47.4)	4 (33.3)	0.48
Superficial punctate keratopathy	7 (36.8)	2 (16.7)	0.42
Corneal epithelial defect	4 (21.1)	0 (0.0)	0.14
Conjunctivitis	3 (15.8)	2 (16.7)	1.00
Blepharitis	2 (10.5)	1 (8.3)	1.00
Madarosis	1 (5.3)	0 (0.0)	1.00
Eyelid malposition	3 (15.8)	0 (0.0)	0.26
Lacrimal drainage obstruction	0 (0.0)	1 (8.3)	0.39
Cataract	3 (15.8)	1 (8.3)	1.00
Anterior segment neovascularization, n (%)	5 (26.3)	0 (0.0)	0.13
Rubeosis iridis	4 (21.1)	0 (0.0)	0.14
Neovascularization of the angle / neovascular glaucoma	5 (26.3)	0 (0.0)	0.13
5-year cumulative incidence of neovascularization, %	29.1	0.0	0.15 §

§ Gray test with death as a competing risk. P values are from the Fisher exact test unless otherwise stated; continuous variables were compared with the Mann–Whitney U test.

This analysis repeats Table 3 within the maxillary sinus, where site, T classification and radiation dose are more nearly balanced. Observation was not: median follow-up was 1784 days in the RADPLAT group and 385 days in the non-RADPLAT group, shorter than the median interval to neovascularization, so the absence of events in the non-RADPLAT group is not evidence of no difference. The third segment of the maxillary artery was infused in 15 patients, the second segment in 3, and the segment was not specified in 1; prophylactic embolization was attempted without success in one additional patient.

Supplementary Table S3. Anterior segment findings observed at the first post-radiotherapy ophthalmological examination versus those detected during subsequent follow-up.

Finding	Observed at the first post-radiotherapy examination	Detected during subsequent follow-up	Total
Any anterior segment finding (per patient)	23	7	30
Superficial punctate keratopathy	14	3	17
Corneal epithelial defect	1	5	6
Conjunctivitis	11	2	13
Blepharitis	3	2	5
Madarosis	1	1	2
Eyelid malposition	1	3	4
Lacrimal drainage obstruction	0	1	1
Anterior segment neovascularization	0	7	7
Rubeosis iridis	0	4	4
Neovascularization of the angle / neovascular glaucoma	0	7	7

A finding was classified as observed at the first post-radiotherapy examination; all other findings were classified as detected during subsequent follow-up.
